# Identification of Restricted Subsets of Mature microRNA Abnormally Expressed in Inactive Colonic Mucosa of Patients with Inflammatory Bowel Disease

**DOI:** 10.1371/journal.pone.0013160

**Published:** 2010-10-05

**Authors:** Magali Fasseu, Xavier Tréton, Cécile Guichard, Eric Pedruzzi, Dominique Cazals-Hatem, Christophe Richard, Thomas Aparicio, Fanny Daniel, Jean-Claude Soulé, Richard Moreau, Yoram Bouhnik, Marc Laburthe, André Groyer, Eric Ogier-Denis

**Affiliations:** 1 INSERM U773, Centre de Recherche Biomédicale Bichat Beaujon, Paris, France; 2 Université Paris 7 Denis Diderot, Paris, France; 3 Service de Gastroentérologie et d'Assistance Nutritive, Hôpital Beaujon, Clichy, France; 4 Service d'Anatomo-Pathologie, Hôpital Beaujon, Clichy, France; 5 Service de Gastroentérologie, Hôpital Xavier Bichat, Paris, France; Emory University, United States of America

## Abstract

**Background:**

Ulcerative Colitis (UC) and Crohn's Disease (CD) are two chronic Inflammatory Bowel Diseases (IBD) affecting the intestinal mucosa. Current understanding of IBD pathogenesis points out the interplay of genetic events and environmental cues in the dysregulated immune response. We hypothesized that dysregulated microRNA (miRNA) expression may contribute to IBD pathogenesis. miRNAs are small, non-coding RNAs which prevent protein synthesis through translational suppression or mRNAs degradation, and regulate several physiological processes.

**Methodology/Findings:**

Expression of mature miRNAs was studied by Q-PCR in inactive colonic mucosa of patients with UC (8), CD (8) and expressed relative to that observed in healthy controls (10). Only miRNAs with highly altered expression (>5 or <0.2 -fold relative to control) were considered when Q-PCR data were analyzed. Two subsets of 14 (UC) and 23 (CD) miRNAs with highly altered expression (5.2->100 -fold and 0.05–0.19 -fold for over- and under- expression, respectively; 0.001<p≤0.05) were identified in quiescent colonic mucosa, 8 being commonly dysregulated in non-inflamed UC and CD (mir-26a,-29a,-29b,-30c,-126*,-127-3p,-196a,-324-3p). Several miRNA genes with dysregulated expression co-localize with acknowledged IBD-susceptibility loci while others, (*eg.* clustered on 14q32.31), map on chromosomal regions not previously recognized as IBD-susceptibility loci. In addition, *in silico* clustering analysis identified 5 miRNAs (mir-26a,-29b,-126*,-127-3p,-324-3p) that share coordinated dysregulation of expression both in quiescent and in inflamed colonic mucosa of IBD patients. Six miRNAs displayed significantly distinct alteration of expression in non-inflamed colonic biopsies of UC and CD patients (mir-196b,-199a-3p,-199b-5p,-320a,-150,-223).

**Conclusions/Significance:**

Our study supports miRNAs as crucial players in the onset and/or relapse of inflammation from quiescent mucosal tissues in IBD patients. It allows speculating a role for miRNAs as contributors to IBD susceptibility and suggests that some of the miRNA with altered expression in the quiescent mucosa of IBD patients may define miRNA signatures for UC and CD and help develop new diagnostic biomarkers.

## Introduction

Ulcerative Colitis (UC) and Crohn's Disease (CD) are two subphenotypes of inflammatory bowel disease (IBD) affecting the intestinal mucosa. UC and CD share similarities such as a chronic relapsing-remitting course and common extra-intestinal manifestations. However, several differences in localization (any part of the gastrointestinal tract -CD- or restricted to the colon -UC), endoscopic appearance and histology support differences in underlying physiopathology.

The current understanding of IBD pathogenesis points out the interplay of genetic, epigenetic and environmental factors in the dysregulated immune response of the intestinal mucosa [1]–[3] where inappropriate control of innate and acquired immunity plays a major role [4].

Long term follow-up stressed that basal colonic lesions extend progressively in more than 50% of UC patients [5]. In CD patients, ileal recurrence involving microscopically quiescent tissues at the time of ileo-colonic resection was reported to reach 73% at one year [6]. These observations suggest that quiescent mucosa of IBD patients display increased susceptibility to inflammation. In this connection, animals models (mice carrying intestinal epithelial cell-specific invalidation of genes involved in the unfolded protein response -XBP1, X-box Protein 1- or essential for embryonic development of the colon -HNF4, Hepatic Nuclear Factor 4) support the notion that epithelial cell dysfunction in the quiescent mucosa can trigger intestinal inflammation [7]–[8]. However the early epithelial disorders that, in pre-inflammatory states, confer susceptibility to uncontrolled mucosal inflammation remain poorly understood.

Strong evidence supports UC and CD as complex genetic disorders with significant overlap and mandates systematic approaches to identify causal molecular events. ***First***, Genome Wide Association Scans (GWAS) [9]–[19] and candidate gene approach [20]–[25] led to the identification of more than 30 susceptibility loci for CD and UC and identified “IBD-specific” gene variants within these loci (*eg. CARD15*, *TNFSF15*, *IL23R*, *ATG16L1*, *IRGM*, *PTPN2*). ***Otherwise***, genome-wide arrays and subtractive hybridization studies identified hundreds of mRNAs with altered expression in non-inflamed [26], [27] and in inflamed [28]–[32] colonic biopsies obtained from UC and CD patients. This provided valuable insights into dysregulated gene expression associated with IBD. In this connection, we hypothesized that dysregulated microRNA (miRNA) gene expression and/or pri-/pre- miRNA maturation may contribute to IBD pathogenesis.

miRNAs are small (∼18–24 nt), non-coding RNAs which, by base-pairing to complementary sequences in the 3′-UTR of selected mRNA targets, prevent protein synthesis either by translational suppression [33], [34] or by degradation of their target mRNAs [35], [36]. miRNAs are regulators of early development, cell fate determination, differentiation, proliferation, apoptosis [37]–[39] and dysregulation of their expression has been involved in various human diseases such as cancer [40]–[44], developmental abnormalities [45], muscular disorders [46] and inflammatory diseases [47]–[51].

In the present paper, ***our first objective*** was to pinpoint alterations in the pattern of miRNA expression in the non-inflamed colonic mucosa of UC and CD patients relative to that of healthy subjects. Indeed, such altered miRNA expression in the quiescent colonic mucosa of IBD patients may account for epithelial dysfunction in the absence of epithelial damage (*eg.* ulcerations) and sensitize the mucosa to severe inflammation and infiltration of immune cells. ***Our second objective*** was to search whether dysregulated expression of several miRNAs may be coordinated and thus contribute to IBD susceptibility.

## Results

In a first series of experiments, miRNA expression was quantified in right and left colon from healthy control subjects. Measuring the abundance of 321 mature human miRNA transcripts by real-time Q-PCR, preliminary analysis (2^−ΔCT^) showed that right and left colon displayed similar patterns of miRNA expression, as exemplified for a subset of miRNAs in Table S1.

In a second series of experiments, miRNA expression was measured by real-time Q-PCR in biopsies from UC and CD patients (Table 1; quiescent and inflamed mucosal tissues, Figure S1). Overall, miRNA expression varied continuously from −11.06 to +20.31 -fold (quiescent and inflamed CD biopsies) and from −7.50 to +18.34 -fold (quiescent and inflamed UC biopsies) when compared to healthy control subjects. However, a careful inspection of the data showed that even under our strictly controlled *(i)* biopsy selection (Figure S2), *(ii)* RT and *(iii)* Q-PCR conditions, miRNA expression levels were variable among patients (Figure S3). Thus, in order to avoid false/erroneous classification of miRNAs as up- and down- regulated in mucosal biopsies of IBD patients, only miRNAs with alterations of expression that fitted stringent thresholds (2^−ΔΔCT^>5-fold and 2^−ΔΔCT^<0.2-fold, respectively) were considered.

**Table 1:** Characteristics of patients with CD or UC.

	Ulcerative Colitis	Cronh's Disease
N° of patients	8	8
Male/Female	5/3	4/4
Age (* y)		
Mean	45.9	37.6
Range	33–57	20–58
Disease duration (y)		
Mean	10.5	8.8
Range	1–21	0.5–23
# Medications (%)		
5 ASA	6 (75)	2 (25)
CS	-	2 (25)
AZA	1 (13)	2 (25)
MTX, IFX	-	1 (13)
CS, 5 ASA	1 (13)	-
None	-	1 (13)

*y, years;

# Medications : CS: steroids/5 ASA: 5 aminosalicylates/AZA: azathioprine/IFX: infliximab/MTX: methotrexate.

### miRNA expression is altered in both UC and CD

In order to check for specific modifications that may account for epithelial cell dysfunction in the quiescent colonic mucosa of IBD patients, we focused on biopsies scored grades 0 and 1 (both grades were observed in healthy controls and in quiescent UC and CD mucosa; Table S2). However, grades 2–4 (inflamed mucosa; *see* Figure S1) were also studied for comparison of both stages of the diseases.

According to our stringent criteria for the selection of miRNAs with altered expression, up- and down- regulations were balanced in UC (45.47% and 54.5%, respectively), whereas up-regulation was predominant (88.2%) in CD.

**Figure 1:**
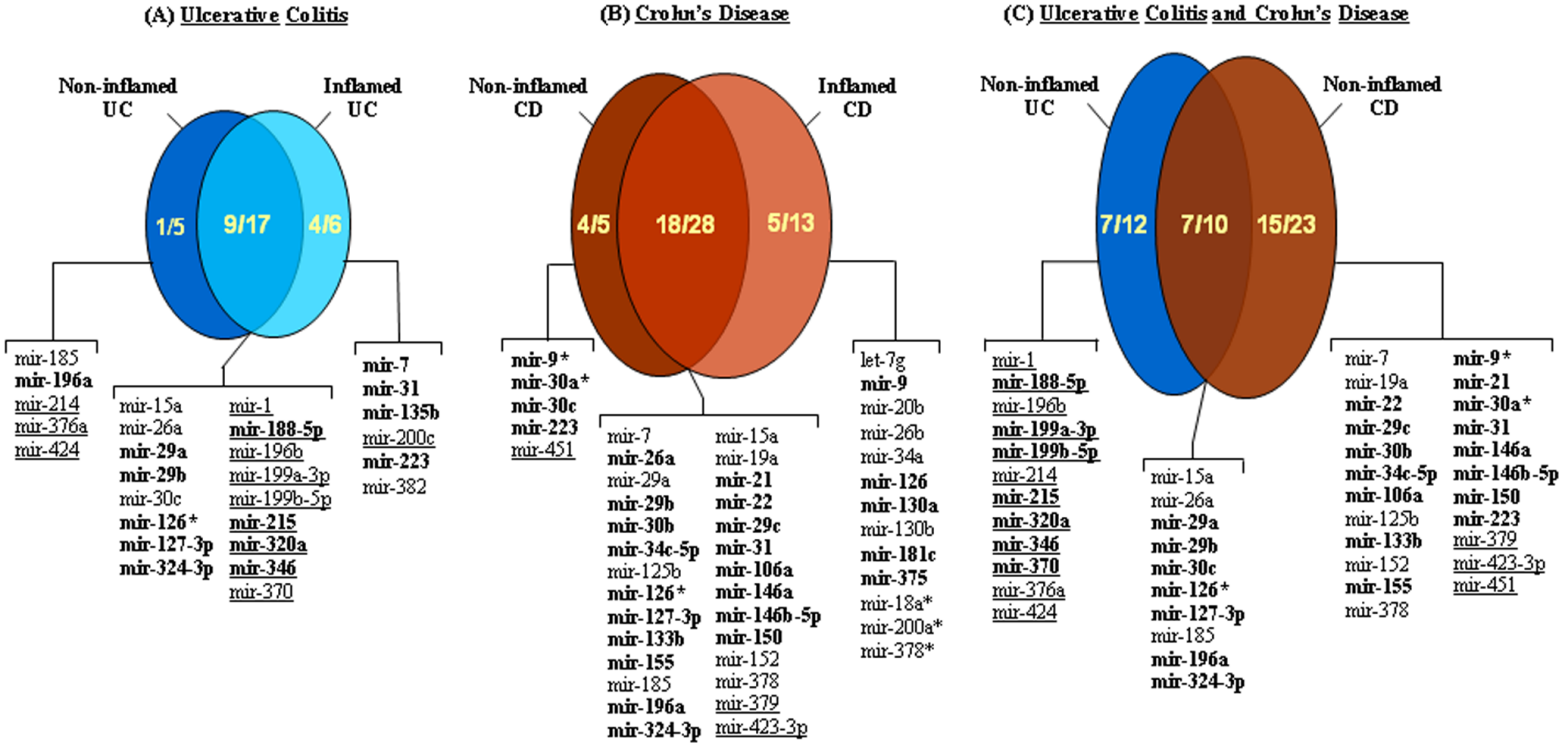
Disease- and stage- specific alterations of miRNA expression. miRNA expression was measured in non-inflamed and inflamed UC and CD tissues and computed *vs.* that measured in healthy controls. The total numbers of miRNAs that were underexpressed and overexpressed in non-inflamed (dark-colored ovals) or inflamed (light-colored ovals) IBD tissues, as well as those that were commonly altered in both states of the disease (intersect between light and dark-colored ovals) were determined. UC (A) or CD (B) tissues were considered independently. (C) miRNAs that were underexpressed and overexpressed in non-inflamed UC (dark-blue) or non-inflamed CD (dark-red), as well as those that were commonly altered in both diseases (intersect between ovals) were determined. Underexpressed miRNAs are underlined. Bolded characters, miRNA with statistically significant dysregulation of expression relative to healthy controls (p≤0.05).

***UC*** **.** 173 miRNAs were expressed above the level of detection (C_T_<35). Of the 22 miRNAs that fit our stringent criteria, only 14 (7 up- and 7 down- regulated) exhibited significant differential expression when non-inflamed UC and healthy control tissues were compared (0.001<p<0.05; non parametric Mann-Whitney test), (Table S3, Figure 1A). With respect to cut-off values and statistical significance the expression of 9 miRNAs was dysregulated in both quiescent and inflamed UC mucosa and that of 1 miRNAs was specifically dysregulated in quiescent UC mucosa (mir-196a).

***CD*** **.** 204 miRNAs were expressed above the level of detection. Of the 33 miRNAs that fit our stringent criteria, only 23 (all up-regulated) exhibited significant differential expression when non-inflamed CD and healthy control tissues were compared (0.002<p<0.05; non parametric Mann-Whitney test), (Table S4, Figure 1B). With respect to cut-off values and statistical significance the expression of 18 miRNAs were dysregulated in both quiescent and inflamed CD mucosa and that of 4 miRNAs were specifically dysregulated in quiescent CD mucosa (mir-9*, mir-30a*, mir-30c, mir-223).

Finally, taking into account cut-off values and statistical significance, we also noticed alterations in miRNA expression specific to inflamed UC or CD tissues (4 and 5 miRNAs, respectively) (Figure 1A, B).

### Common and specific alterations in miRNA expression in UC and CD

With respect to cut-off values and statistical significance 8 miRNAs shared common altered expression in non-inflamed CD and in non-inflamed UC (Figure 1C, Table S5), of which 6 (all but mir-30c, and mir-196a) were also overexpressed both in inflamed UC and in inflamed CD biopsies.

**Figure 2:**
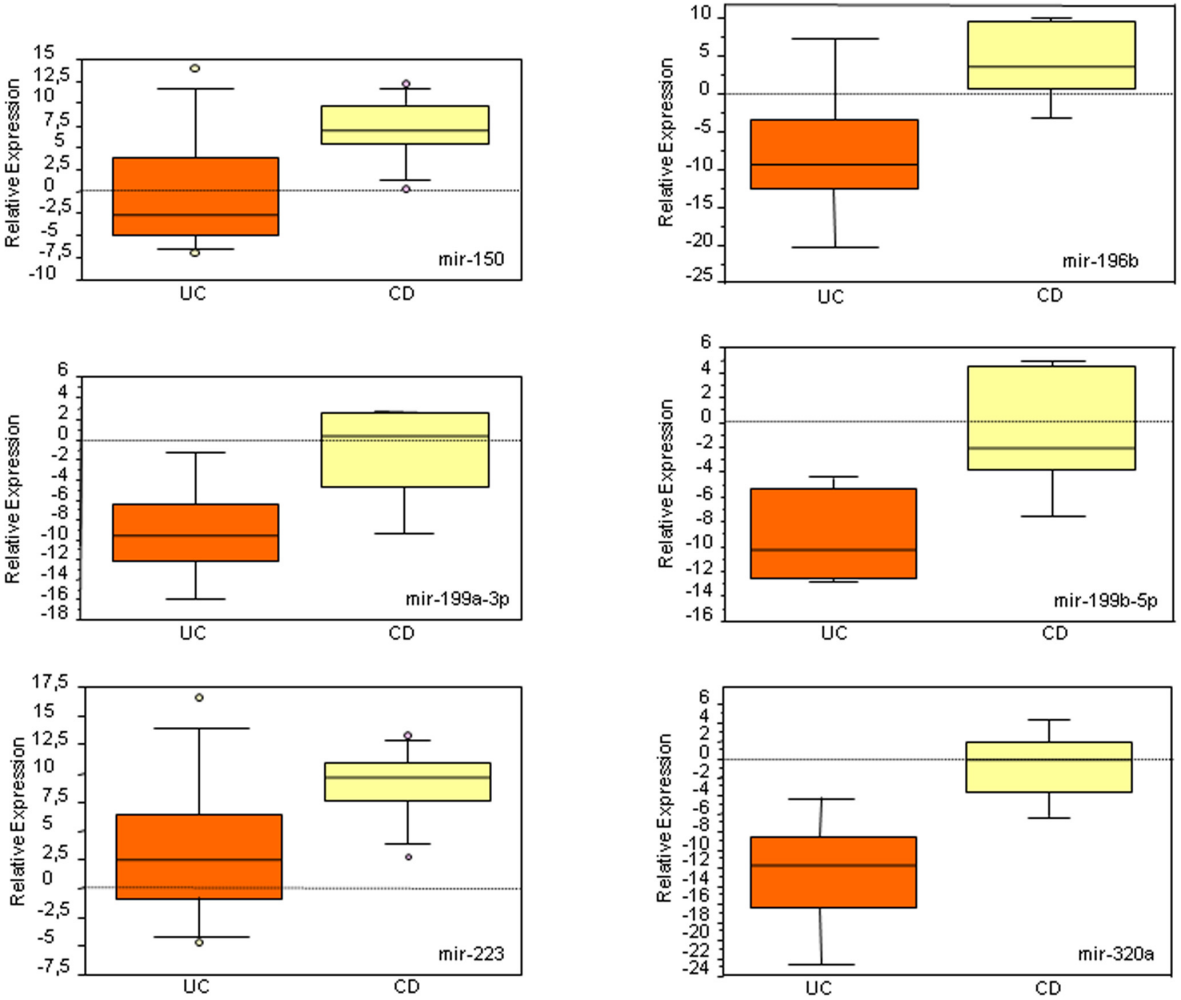
miRNAs with differentially altered expression in non-inflamed UC and CD tissues : Box-whisker plot analysis. miRNA expression was measured in non-inflamed colonic mucosa obtained from UC and CD patients (8 patients/IBD) and computed *vs.* that measured in healthy controls. Data corresponding to 6 miRNAs (mir-150, mir-196b, mir-199a-3p, mir-199b-5p, mir-223 and mir-320a) with statistically different alteration of expression in UC and CD mucosal tissues are presented as box-whisker plots [78] (box, 25–75%; whisker, 10–90%; line, median); p<0.05.

On the other hand, the expression of 6 miRNAs was statistically different in non-inflamed colonic biopsies of UC and CD patients (mir-150, *p* = 0.0273; mir-196b, *p* = 0.0472; mir-199a-3p, *p* = 0.0472; mir-199b-5p, *p* = 0.0283; mir-223, *p* = 0.0357 and mir-320a, *p* = 0.0163; non-parametric Mann-Whitney test) (Figure 2). These miRNAs, and an additional selection of 9 miRNAs (selected in an unsupervised manner using the GenePattern “ComparativeMarkerSelection” module) (Owing to patent pending the identity of these miRNA is not disclosed in the manuscript) were tested for their ability to discriminate between UC and CD. Classification was performed with a supervised algorithm (GenePattern “KNNXValidation” module). Based on the clinical classification of our panel of patients as UC or CD, the selection of 15 miRNAs was able to predict 15/16 patients in their true class (Table 2).

**Table 2:** Achievement of patient's class (UC or CD) prediction using the selection of 15 miRNAs.

Patient	True Class		Predicted Class	Confidence	Correct ?	
	Initial Diagnostic	Reassessment After Clinical Follow-up			Relative to Initial Diagnostic	After Clinical Follow-up/Reassessment
CD_Quiescent_28	Cd	Not modified	Uc	1	false	
CD_Quiescent_102	Cd	Not modified	Cd	1	true	
CD_Quiescent_111	Cd	Not modified	Cd	1	true	
CD_Quiescent_120	Cd	Not modified	Cd	1	true	
CD_Quiescent_130	Cd	Not modified	Cd	0,7894	true	
CD_Quiescent_137	Cd	Not modified	Cd	1	true	
CD_Quiescent_158	Cd	Not modified	Cd	1	true	
CD_Quiescent_160	Cd	Not modified	Cd	1	true	
UC_Quiescent_107	Uc	Not modified	Uc	1	true	
UC_Quiescent_125	Uc	Not modified	Uc	0,8144	true	
UC_Quiescent_121	Uc	Not modified	Uc	1	true	
UC_Quiescent_114	Uc	Not modified	Uc	0,5339	true	
UC_Quiescent_109	Uc	Not modified	Uc	0,5508	true	
**UC_Quiescent_13**	**Uc**	**Cd**	**Cd**	0,6229	**false**	**true**
UC_Quiescent_15	Uc	Not modified	Uc	0,6415	true	
UC_Quiescent_132	Uc	Not modified	Uc	0,5176	true	

The 6 miRNAs that displayed significantly distinct alteration of expression in non-inflamed colonic biopsies of UC and CD patients and 9 additional miRNAs, which were selected in an unsupervised manner making use of the GenePattern “ComparativeMarkerSelection” module, were tested for their putative use as “biomarkers”. The test was carried out using the “KNNXValidation” module computed on line from the GenePattern server. **Of note**, patient “UC_Quiescent_13”, initially classified as UC on the basis of clinical criteria, was predicted as CD using our selection of 15 miRNAs. Interestingly its clinical follow-up for several years led to the reassessment of its clinical classification as CD.

Altogether, these data unambiguously show that altered miRNA expression pre-exists in non-inflamed UC and CD mucosa.

### Concerted regulation of miRNA expression in UC and CD

We then sought whether the altered levels of miRNA noticed in both quiescent UC and CD colonic mucosa could be accounted for by coordinated regulation(s) of miRNA expression. *In silico* clustering was achieved in an unsupervised manner using a K-Means algorithm, the expression data being partitioned into 20 distinct computational clusters. Interestingly, 7/8 miRNAs overexpressed both in non-inflamed UC and CD tissues (mir-26a, mir-29a, mir-29b, mir-30c, mir-126*, mir-127-3p, mir-324-3p), localized on different chromosomes, were assigned to a single computational cluster (cluster #7) when examining UC data, and to two such clusters (cluster #7: mir-26a, mir-30c, mir-127-3p, mir-324-3p and cluster #13: mir-29a, mir-29b, mir-126*) when CD data were inspected (Table 3). Moreover, five of these miRNAs (mir-26a, mir-29b, mir-126*, mir-127-3p, mir-324-3p) were also assigned to a single computational cluster when inflamed UC (cluster #7) and CD (cluster #13) data were classified. This suggested common regulation of expression for mir-26a, mir-29b, mir-126*, mir-127-3p and mir-324-3p.

**Table 3:** Concerted regulation of expression of miRNAs in non-inflamed and inflamed CD and UC tissues.

Overexpressed miRNA			
UC		CD	
Non-inflamed	Inflamed	Non-inflamed	Inflamed
**Cluster #7 :**	**Cluster #7 :**	**Cluster #7 :**	**Cluster #13 :**
mir-15a	mir-7	mir-7	**mir-26a**
**mir-26a**	**mir-26a**	**mir-26a**	**mir-29b**
mir-29a	mir-29a	mir-30b	mir-126*
**mir-29b**	**mir-29b**	mir-30c	mir-155
mir-30c	mir-31	**mir-127-3p**	**mir-127-3p**
mir-126*	mir-126*	mir-155	mir-185
**mir-127-3p**	**mir-127-3p**	mir-223	mir-196a
**mir-324-3p**	mir-135b	**mir-324-3p**	**mir-324-3p**
	**mir-324-3p**		mir-378
		**Cluster #13 :**	
		mir-29a	
		**mir-29b**	
		mir-126*	
		mir-196a	

Alterations in miRNA expression (8 UC, 8 CD patients) were clustered using a K-Means algorithm (computed on-line on the GenePattern server), independently in each IBD and for each state of the disease. Clusters that encompass several miRNAs with similarly up-regulated expression are highlighted (bold characters).

### Chromosomal localization of miRNA genes with altered expression

The chromosomal distribution of miRNA genes with altered expression in UC and CD mucosa was not even. Indeed, 9 chromosomes (1, 5, 9, 11, 14, 15, 17, 19 and X) housed ≥4 and up to 12 miRNA genes with dysregulated expression (overall: ∼70% of such miRNAs genes) (Table S6, Figure S4). The chromosomal loci where miRNA genes with dysregulated expression are localized encompass either one, two (miRNA duplexes) or more (miRNA clusters) distinct miRNA genes.

Interestingly, it could be observed that several miRNAs mapped within acknowledged IBD susceptibility loci (IBD-2, 3, 5 and 6), or colocalized with genetic variations identified in several GWAS studies that *(i)* account for part of the overall genetic susceptibility to CD and *(ii)* contribute to UC pathogenesis (Figure S5, Table S7). None mapped with IBD susceptibility loci 1, 4, 7, 8 and 9.

Otherwise, one chromosomal miRNA cluster (on chromosome 14q32.31) and several miRNA duplexes (6q13, 7q32.3, 9q34.11–q34.3, 15q26.1; 17p13.1–p13.3, 22q11.21, Xq26.2) were identified that map on chromosomal regions that have not been previously reported as IBD-susceptibility loci (Figure S5, Table 4). Interestingly, in the majority of loci, alterations of miRNA expression were observed in quiescent UC and CD tissues.

**Table 4:** Compilation of the sub-chromosomal regions where two or more miRNA genes with altered expression colocalize.

Chromsome	miRNA		Alteration of Expression			Duplex/Cluster
	Gene_Id	Locus	IBD_Type	Disease state	+/−	
6	30c-2	6q13	CD/UC	Quiescent/Quiescent	+	miRNA Duplex
	30a*	6q13	CD	Quiescent	+	
7	29a	7q.32.3	CD/UC	Both/Both	+	miRNA Duplex
	29b-2	7q.32.3	CD/UC		+	
9	199b-5p	9q34.11	UC	Both	−	miRNA Duplex
	126	9q34.3	CD	Inflamed	+	
	126*	9q34.3	CD/UC	Both/Both	+	
14	127-3p	14q32.31	CD/UC	Both/Both	+	miRNA Cluster
	370	14q32.31	UC	Both	−	
	382	14q32.31	UC	Inflamed	+	
15	7-2	15q26.1	UC	Inflamed	+	miRNA Duplex
	9-3	15q26.1	CD	Inflamed	+	
17	22	17p13.3	CD	Both	+	miRNA Duplex
	324-3p	17p13.1	CD/UC	Both/Both	+	
22	185	22q11.21	CD	Both	+	miRNA Duplex
	130b	22q11.21	CD	Inflamed	−	
X	106a	Xq26.2	CD	Both	+	miRNA Duplex
	20b	Xq26.2	CD	Inflamed	+	

Compilation of chromosomes and bands where colocalize 2 (miRNA Duplex) or more (miRNA Cluster) miRNA genes with altered expression relative to healthy controls in UC or CD tissues. Gene_Id, miRNA gene identification number; Locus, chromosomal band where the miRNA gene is localized; Quiescent, non-inflamed; Both, non-inflamed and inflamed; +/−, +: overexpression; −: underexpression.

### Alteration of miRNA expression: *in silico* characterization of target transcripts

Identification of a subset of 8 miRNAs that share common regulated overexpression in both UC and CD (*see* Table S5) could represent the first step towards the identification of regulatory networks, the dysregulation of which could be involved in the pathophysiology of IBD. *In silico*, 4094 genes (372 strictly down-regulated genes) stand as putative targets for these miRNAs.

**Figure 3:**
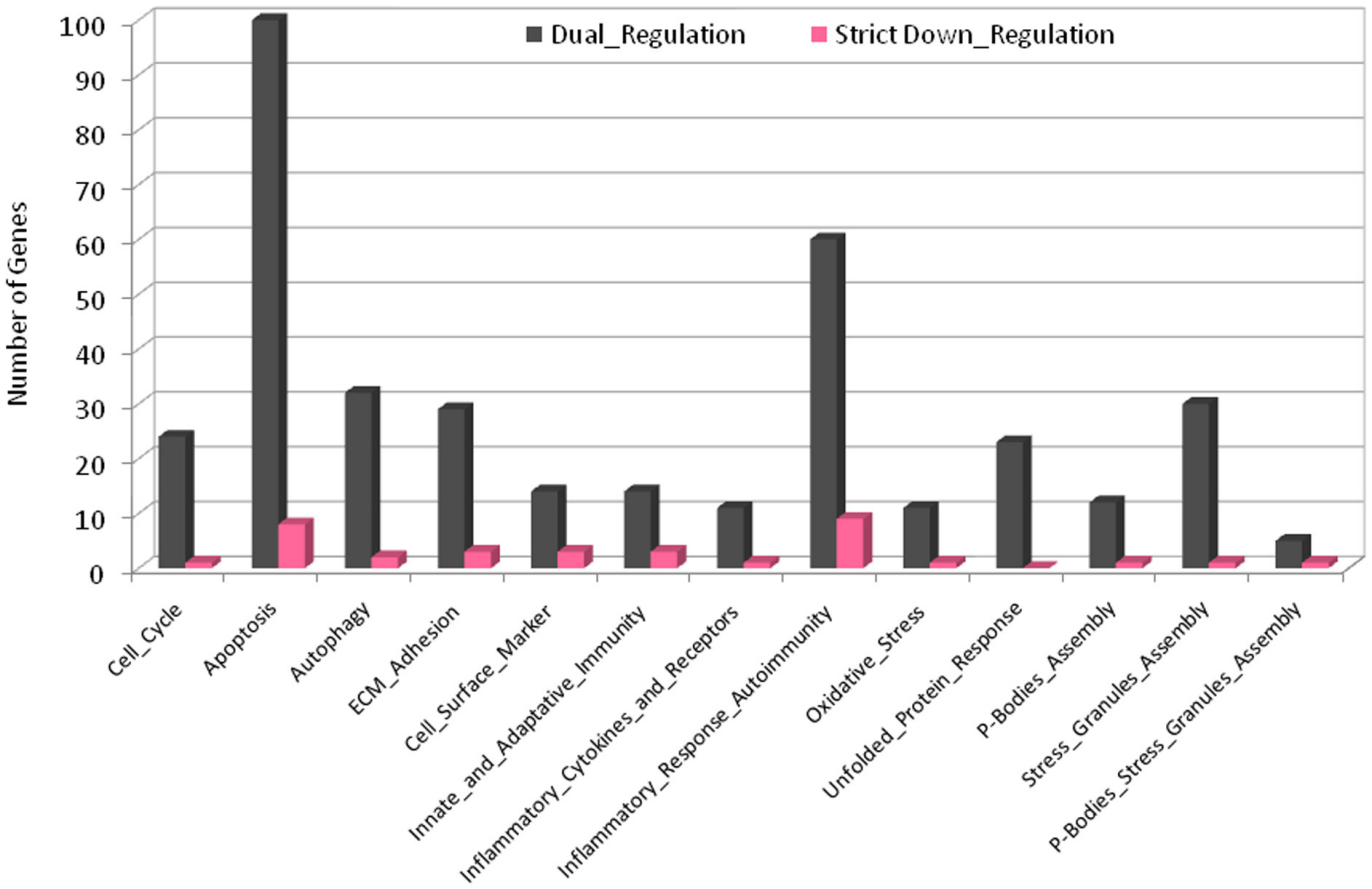
Alteration of miRNA expression in the colonic mucosa of UC and CD patients: *in silico* characterization of target transcripts. The exhaustive list of genes which are putatively targeted by the subset of 8 miRNAs that share common dysregulated expression both in quiescent UC and in quiescent CD was downloaded from the PITA catalog of predicted human microRNA targets (http://genie.weizmann.ac.il/pubs/mir07/mir07_data.html). The algorithm makes use of the parameter-free model for miRNA-target interaction described by Kertesz *et al.* [75]. The total number of genes involved in each single biological process is computed. Strict down regulation (light purple) stands for genes, the 3′-UTR of which interacts only with up-regulated miRNA(s).

Exploring the molecular functions associated to these gene products using The Gene Ontology, GeneCards and GeneNote data bases, we found associations to several biological processes (Figure 3). These include *(i)* cell proliferation (Cyclins D1, D2 and E1, PCNA, CDKs 6 and 8, GADD45A, RB1), *(ii)* apoptosis (BCL2, Caspase 2, C/EBP β,γ, DAPK, FOXO3, PTEN), *(iii)* autophagy (ATG 4a, 5 and 9a, Beclin-1, CDKN1B, IFNγ), *(iv)* extracellular matrix organization, cell adhesion and cell surface marker gene expression (COL(1,11,12,15,16)A1, Integrin-α_1,2,3,5_, β_1,3_, Laminin γ_1_, MMPs 13 and 16, Keratin 5, NCAM1), *(v)* oxidative stress (GPX4, OXR1, OSXR1), *(vi)* the unfolded protein response (HSPA5, HSPA2, SERP1, XBP-1, EIF2AK3, ETF1) *(vii)* innate and adaptive immunity (IL1A, IL10, IL1R1, IL6R, IRAK2, p40^phox^, TLR10, CXCL2, 12 and 14, CXCR4, NFATC3 and C4, PREX1). In addition, several of these genes are acknowledged IBD-susceptibility genes or are localized at replicated risk loci identified by GWAS (*eg.* ATG16L1, IL10, IL12B, JAK2, ARPC2, PTGER4, ZNF365, NKX2-3, PTPN2, PTPN22, C11orf30, ORMDL3, STAT3 (Table S8).

## Discussion

In the present study, we have addressed the question of whether altered miRNA expression in quiescent UC and CD mucosa may be relevant to IBD pathogenesis. Our data allowed two major conclusions.

***First***. Alteration of miRNA expression was not confined to inflamed (grades 2–4), but preexisted in non-inflamed (grades 0 and 1) mucosa. Applying strictly controlled RT and real-time Q-PCR protocols and stringent cut-off values (>5-fold or <0.2-fold *vs.* healthy individuals), we identified 14 and 23 miRNAs with dysregulated expression in non-inflamed UC and CD biopsies, respectively, of which 8 were similarly dysregulated both in non-inflamed UC and CD biopsies. Our observation that mir-26a and 29a are up-regulated in quiescent UC mucosa has also been reported by Wu *et al.* [51]. In contrast, the other miRNAs (9/10, mir-629 was not tested in our screen) which were found to be up- (*eg.* mir-21, mir-126 and Let-7f) or down- (*eg.* mir-19b) regulated in [51] displayed only slight alterations in relative expression that did not match the stringent selection criteria we applied in our study. This suggests *(i)* that the discrepancies between both studies may be explained either by the differential sensitivity of the methods used for initial screening (microarray *vs.* real-time Q-PCR) and/or rather by the drastic cut-off value (>5-fold or <0.2-fold) we used to state altered miRNA expression in the present study and *(ii)* that the overlap in the alteration of miRNA expression observed in our study and in that reported by Wu *et al.* [51] may not have occurred only by chance. As far as we are aware, alteration of miRNA expression in non-inflamed CD colonic biopsies has not yet been reported.

Importantly, despite *(i)* the choice of a drastic cut-off that takes into account the variability in miRNA expression between IBD patients and *(ii)* the limited number of subjects kept for analysis in the present study, we could select miRNAs highly and significantly dysregulated in IBD relative to healthy controls. Interestingly, comparison of non-inflamed to inflamed tissues showed significant overlap in the alteration pattern of miRNA expression both in CD patients (this study) and UC (this study, [51]).

Altogether, these results support the notion that dysregulation of miRNA expression pre-exists in the quiescent colonic mucosa of UC and CD patients and may play a key role in the sensitization of the quiescent mucosa to environmental factors and/or to IBD inducers (*ie.* commensal flora), and *in fine* the onset and/or relapse of inflammation. Furthermore, they suggest that quiescent UC and CD mucosa already has distinct miRNA signatures which are not associated with significant variations in cellular contexts. Indeed, the quiescent colonic mucosa of IBD patients and that of healthy subjects were almost similar (grades 0 or 1 in both cases).

Since significant overlap was observed in the alteration of miRNA expression in quiescent UC and CD mucosa, our results also imply that several common molecular mechanisms may underlie the UC and CD pathogenic processes. Furthermore, alteration of miRNA expression in quiescent IBD tissues is consistent with the notion that genetic variants that result in differential gene expression (*eg.* that of regulatory molecules such as miRNAs) as well as mutations in the open reading frame are expected to contribute to multifaceted diseases.

In this connection, one major drawback in investigating the dysregulation of miRNAs and of protein-coding genes expression in IBD tissues is related to cell type variations between samples (*eg.* inflamed *vs.* quiescent mucosa and normal healthy tissue). Indeed, inflamed mucosal tissue is characterized by a decreased number of epithelial cells, concomitant with an increased infiltration of inflammatory cells. This bias was taken into account in some genome wide cDNA microarray studies [26] but not in others [31]. For instance, decreased MICA (a gene expressed in intestinal epithelial cells) transcript expression was reported in inflamed CD [31] whereas flow cytometry and immuno-histochemistry identified strong MICA overexpression in intestinal epithelial cells of macroscopically involved areas of CD patients [52]. Similarly, it could be anticipated that the decreased level of mir-192 expression in inflamed mucosa of UC patients [51] may depend on cell-type heterogeneity between non-inflamed and inflamed mucosal tissues rather than on decreased gene expression (of note, in our study the slight decrease in mir-192 expression did not match our selection criteria in inflamed UC mucosa). Thus, the increase in MIP2α expression observed in [51] could be miRNA-independent and accounted for by increased TNFα secretion by immune infiltrating cells.

Finally, starting with a wide screen of 321 miRNAs, we could define *(i)* a selection of 8 miRNAs relevant in defining quiescent IBD *vs.* healthy mucosa and *(ii)* a distinct subset of 15 miRNAs (Patent pending) that allows discriminating between non-inflamed UC and CD colonic mucosa and may define specific biomarkers relevant for UC and CD. Indeed on the basis of our panel of 16 patients, this selection of 15 miRNAs was able to predict 15/16 patients (94%) as UC or CD correctly. Such biomarkers may prove helpful as diagnostic tools of minimal invasivity (*eg.* for pediatric patients, incomplete colonoscopy) and as guidelines for surgical decisions. It can also be anticipated that miRNA signatures could be associated with different IBD profiles as prognostic biomarkers. This is out of the scope of the present study and deserves further studies on a larger cohort of patients.

***Second.*** miRNAs play a major role in regulating coding-gene expression at the transcript and/or translational levels [34], [36]. In this connection, we would like to emphasize that our study is the first one that reports the mapping of several miRNA genes with altered expression in quiescent UC and CD mucosa *(i)* at acknowledged IBD loci [53]–[55] or *(ii)* at loci conclusively associated with CD [11], [16] } or UC [12], [13], [19] by GWAS studies. In this connection, we should like to emphasize that the co-localization of miRNA genes with dyregulated expression at chromosomal loci associated with IBD susceptibility does not occur only by chance. Indeed, our computations show that 1 miRNA gene (out of 321 tested) would be expected to be localized by chance in the vicinity of the 50 loci reported in [11], [12], [16]–[19] where 14 miRNA genes (14-fold more) with altered expression in quiescent UC and CD tissues map. In addition, even if 8 miRNA genes could map by chance within IBD susceptibility loci 1, 4, 7, 8 and 9, no miRNA genes with altered expression in quiescent UC and CD tissues were localized in these chromosomal regions (although they encompass a total of 18 miRNA genes, the expression of which is not altered in IBD).

On this basis, we speculate that in addition to mutational events, IBD susceptibility might result from dysregulated miRNA expression in intestinal mucosa and to subsequent alteration of miRNA-dependent regulation of gene expression; consistent with the notion that not only allele variation, but also the alteration of regulatory processes that result in differential gene expression may contribute to multifaceted diseases.

Furthermore, our study characterizes band 14q32.31 as a cluster of 3 miRNA genes with altered expression in IBD. With the exception of mir-382, these miRNAs display altered expression in quiescent UC (mir-127-3p, mir-370) or in quiescent CD (mir-127-3p) mucosa. These miRNA genes are intergenic and constitute at least two distinct transcription units (mir-127 and mir-370). Alteration of miRNA expression within this sub-chromosomal region does not result from the overall chromosomal environment since *(i)* only the expression of 3 (UC) and 1 (CD) miRNAs was altered out of 38 localized within a DNA stretch of 44.74 kbp at 14q32.31, *(ii)* expression was either increased (mir-127-3p, CD/UC; mir-382, UC) or decreased (mir-370, UC) and *(iii)* expression was altered either in non-inflamed or in inflamed or in both states of the diseases. We speculate band 14q32.31 may represent a new, yet undefined IBD-susceptibility locus; this remains to be established and will be the subject of future studies.

Finally, the tight coordinated regulation of mir-26a, mir-29b, mir-126*, mir-127-3p and mir-324-3p (which genes are widespread on several chromosomes) in non-inflamed UC and CD mucosa also suggests that alteration of miRNA expression do contribute to the physiopathology of IBD. Interestingly, such concerted regulation of expression correlates with related biological functions. For instance, these miRNAs have been demonstrated to play roles either in cell cycle regulation, or in tumorigenesis in a broad spectrum of solid tumors (mir-26a, mir-29b, mir-127-3p and mir-324) [56]–[61], in the regulation of epithelial-mesenchymal transition and invasiveness (mir-126*) [62], [63] or in the control of apoptosis (mir-29b and mir-126*) [64], [65], in line with the higher than spontaneous occurrence of colorectal cancer (5-10%) in IBD patients. Of note, a recent study has reported that the mir-29 family of miRNAs regulates intestinal membrane permeability [66]. This observation should be connected with the increased gut permeability observed in IBD patients [67].

*In silico* studies emphasized that the transcripts targeted by the 8 miRNAs which share common overexpression in the quiescent colonic mucosa of both UC and CD patients correspond to genes that are involved/implicated in several cellular processes (*eg.* proliferation, apoptosis, autophagy, extracellular matrix organization, cell surface marker gene expression, oxidative stress, unfolded protein response, innate and adaptive immunity). Several of these genes stand as acknowledged IBD susceptibility genes or as genes of interest localized at convincingly replicated risk loci identified by GWAS (*eg.* ATG16L1, IL10, IL12B, JAK2, ARPC2, PTGER4, ZNF365, NKX2-3, PTPN2, PTPN22, C11orf30, ORMDL3, STAT3). However, an exhaustive identification of the genes targeted by UC- and/or CD- associated miRNAs (*eg.* common to or distinct between UC and CD), the demonstration of their actual regulation by miRNAs and the investigation of their influence on intestinal inflammation in experimental models of colitis is far beyond the scope of this paper and will be the subject of future studies.

Our study supports miRNAs as crucial players in the onset and/or relapse of inflammation from quiescent mucosal tissues in UC and CD patients. It further highlights their putative role as contributors to IBD susceptibility and thus will help unravel the mechanisms (either distinct or shared between UC and CD) involved in relapsing (*eg.* identification of key targets and of gene networks). Finally, they may help develop new biomarkers to distinguish UC and CD at early stages.

## Materials and Methods

### IBD patients and controls

Colonic pinch biopsies were obtained in the course of endoscopical examination of patients with mild to severe CD and UC and of healthy control subjects undergoing screening colonoscopies (Table S2 for clinical details). Colonic biopsies were punctured from 24 CD, 18 UC and 19 healthy controls (*see* Figure S2). However, for the reasons outlined below (*see* paragraphs “Histopathological analyses” and “RNA isolation”) and in Figure S2, the biopsies collected from some patients were not included in the study. Overall, expression of mature miRNAs was studied in inactive colonic mucosa of 8 patients with UC, 8 patients with CD and in 10 healthy control mucosa.

The diagnosis of UC and CD adhered to the criteria given by Lennard-Jones [68]. Clinical disease activity for CD and UC was assessed according to the Harvey-Bradshaw [69] and to the Colitis Activity (CAI) [70] indexes, respectively. In each IBD patient, endoscopically non-inflamed and inflamed areas of colonic tissue were punctured (5 biopsies/area). Non-inflamed and inflamed areas for biopsy collection were separated by more than 20 cm along the colon. Three biopsies from each area were allocated for immediate RNAlater™ immersion then snap frozen and stored in liquid nitrogen, and two were set apart for histopathological examination. In healthy controls, 5 biopsies were punctured both in right and left colon and processed as above. The protocol was approved by the local Ethic Committee (Comité de Protection des Personnes -CPP- Ile de France IV n°2009/17 and AFFSSAPS D91534-80) and written informed consent was obtained from all patients.

### Histopathological analyses

Biopsies were routinely stained with haematoxylin and eosin. Histological assessments of mucosal damage and inflammatory cells infiltration were graded by the same expert gastrointestinal pathologist (DCH) using a score previously validated to characterize the colonic involvement of both UC and CD [71]. Grades were as follows: 0, no evidence of inflammation (normal mucosa); 1, oedema and mild infiltration in the *lamina propria*; 2, crypt abscess and inflammation in the *lamina propria*; 3, severe inflammation with destructive crypt abscess and 4, severe inflammation with active ulceration. Grades 0–1 were considered as quiescent (or non-inflamed) mucosa. Grades 2–4 corresponded to various degrees of inflammation of the mucosa and were considered as active disease (Figure S1). Alterations in miRNA expression were studied following this histological dichotomy (0–1 *vs.* 2–4). IBD patients selected for miRNA analysis had both histologically assessed quiescent and inflammatory samples. 7 CD patients and 3 UC patients were excluded because their endoscopically quiescent colonic mucosa was classified as histologically active. 1 control patient with lymphocytic colitis was excluded (Figure S2).

### RNA Isolation

Total RNA was extracted with TRIzol® Reagent (Invitrogen) then quantified using a ND-1000 NanoDrop spectrophotometer (NanoDrop Technologies) and purity/integrity was assessed using disposable RNA chips (Agilent RNA 6000 Nano LabChip kit) and an Agilent 2100 Bioanalyzer (Agilent Technologies, Waldbrunn, Germany). Only RNA preparations with RIN≥7 were further processed for analysis of miRNA expression. Nine CD, 7 UC and 8 controls with RNA preparations of insufficient purity (RIN<7) were excluded. Finally 8 CD, 8 UC and 10 controls with stringent homogeneity in histological assessment and RNA quality were selected for analysis (Figure S2).

### Reverse-Transcription and Real-Time Q-PCR

The Human Early Access Release Kit (based on miRBase v 9.2; TaqMan® MicroRNA Assay; Applied Biosystems) designed to quantify 321 mature human miRNAs was used. cDNA was generated from 10 ng of total RNA using miRNA-specific stem-loop RT primers. Real-Time Q-PCR assays were performed according to the manufacturer's instructions using aliquots of cDNA equivalent to ∼1.3 ng of total RNA and were run in a Light Cycler 480 (Roche Diagnostics).

***Normalization of Real-Time Q-PCR results.*** Several RNAs (U6, U24, U48 and S18) were tested as putative standards and U6 (an ubiquitous small nuclear RNA) (Primer for U6 were included in the TaqMan® MicroRNA Assay) was found the most reliable. Expression of miRNAs was computed relative to that of U6 and a comparative threshold cycle method (2^−ΔΔCT^) [72] was used to compare non-inflamed and inflamed IBD tissues with healthy controls. Since the abundance of mature miRNA transcripts was expressed relative to that of the reference gene U6, we have checked that PCR efficiencies were identical for test (miRNAs) and reference (U6) transcripts, so that the comparison be accurate (*see* the MIQE guidelines; [73]).

CT, the fractional cycle number at which the amount of amplified target reaches a fixed threshold, was determined (default threshold settings were used in all instances). The cycle number above which the CT was considered as a false positive (cycle cut-off point) was set up at 35, as already argued in the literature dealing with limits of detection in Real-Time Quantitative- RT-PCR [74] ( reviewed in [73]). −ΔΔCT was calculated as follows:
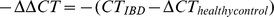
where
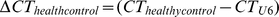
And
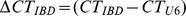



***Determination of cut-off values for miRNA over- and under- expression.*** Relative miRNA expressions (2^−ΔΔCT^) were computed as their log transformed (10×log_10_) values (after such computation up- and down- regulations were expressed as positive and negative values, respectively), and their means and standard deviations (SD_miRNA_) were calculated independently for every miRNA, in each IBD at each stage of the disease. Box-whisker plots analysis of SD_miRNA_ pointed highly dispersed values among patients (Figure S3). Overall, when the data gathered from the two series of patients were considered (8 UC, 8 CD; non-inflamed and inflamed areas of colonic tissues), a mean value of 6.3±1.4 (mean_Disp_ ± SD_Disp_) was calculated for the SD_miRNA_ values. Given such variation in relative miRNA expression, only those with mean 10×log_10_2^−ΔΔCT^>7 and <−7 (±|mean_Disp_+0.5 SD_Disp_|) were considered as up- (2^−ΔΔCT^>5-fold) and down- (2^−ΔΔCT^<0.2-fold) regulated relative to healthy.

### *In silico* prediction of miRNA targets

Exhaustive human miRNA targets were predicted using a parameter-free model for miRNA-target interaction. This model computes the difference between the free energy gained from the formation of the miRNA-target duplex (ΔG_duplex_) and the energetic cost of unpairing the target (and proximal flanking sequences) to make it accessible to the miRNA (ΔG_open_) [75].

We made use of the PITA catalog of predicted human microRNA targets (http://genie.weizmann.ac.il/pubs/mir07/mir07_data.html). The seed parameter settings described in Kertesz *et al.* [75] were followed: seeds of 8 bp in length, beginning at position 2 of the miRNA were chosen, seed conservation being set at 0.9. No mismatches or loops were allowed, but a single G∶U wobble was permitted. In genes missing a 3′ UTR annotation, 800 bp downstream of the annotated end of the coding sequence were used as the predicted 3′ UTR. Flanks of 3 and 15 bp upstream and downstream the miRNA target, respectively, were considered in the computation of ΔG_open_.

In some instances (mir-126*) predictions from the miRBase database (miRBase Targets Release Version v5; http://microrna.sanger.ac.uk/cgi-bin/targets/v5/mirna.pl?genome_id=2964) were downloaded. These predictions combine the miRanda algorithm and the conservation of miRNA binding sites in orthologous transcripts from at least two species (http://microrna.sanger.ac.uk/targets/v5/info.html for details) [76].

### Biological functions of the *in silico* predicted miRNA targets

We made use of the Gene Ontology (http://www.geneontology.org), GeneCards (http://www.genecards.org/) and GeneNote (http://bioinfo2.weizmann.ac.il/cgi-bin/genenote/home_page.pl) databases to document the biological functions of the genes that were predicted to be targeted by miRNAs with altered expression in quiescent UC and CD tissues (*see* Table S8).

### Statistical analysis

Unpaired groups of values were compared according to the non-parametric Mann-Whitney test. Statistical significance was set at p≤0.05.

miRNA which shared closely related expression patterns were grouped according to K-means clustering [77] computed on line from the GenePattern Server. The specified number of clusters was set at 20.

When supervised class (UC or CD) prediction of individual patient's data was tested, we used a K Nearest Neighbors Classification algorithm with Leave-One-Out Cross-Validation (GenePattern “KNNXValidation” module). The class predictor was uniquely defined by the initial set of patients and marker miRNAs. The classifications were tested in leave-one-out cross-validation mode by iteratively leaving one sample out, training a classification on the remaining data and testing on the left out sample.

## Supporting Information

Figure S1 Histological grading of disease activity in colonic biopsies. Hematoxylin and eosin staining of biopsies from non-inflamed and inflamed colonic mucosa (see Materials and Methods for details on histological grading). Note the progressive loss of intestinal epithelium with increasing grade of the disease (2–4) and the concomitant increase in the severity of inflammation/infiltration. Magnification, ×100.
(0.35 MB TIF)

Figure S2 Flow chart of sample selection. In order to exclude any bias in homogeneity among samples, biopsies of patients with endoscopically quiescent, but histologically active colonic mucosa were excluded. The histological dichotomy (grades 0–1 vs. 2–4) was strictly followed to study the alteration in miRNA gene expression. Similarly, 1 control patient with lymphocytic colitis was excluded. In all cases, RNA preparations of low integrity (RIN<7) were discarded.
(0.09 MB TIF)

Figure S3 Alteration of miRNA gene expression in non-inflamed and inflamed UC and CD tissues : Box-whisker plot analysis of standard deviations. miRNA expression was measured in non-inflamed and inflamed colonic mucosa obtained from patients with UC and CD (8 patients/IBD) and computed vs. that measured in healthy controls. The mean and standard deviation (SDmiRNA) of relative miRNA expression were then calculated for every miRNA, independently in each IBD for each state of the disease. SDmiRNA were then plotted as box-whisker plots (box, 25–75%; whisker, 10–90%; line, median) (1). 1 Tukey, J. W. (1977) in Exploratory Data Analysis (Addison-Wesley, Reading, MA), pp. 39–43.
(0.06 MB TIF)

Figure S4 Overview of the chromosomal distribution of miRNA genes with altered expression in IBD tissues: The total number of miRNA genes with altered expression was determined by chromosome. Negative and positive ordinates stand of over and under -expression, respectively.
(0.10 MB TIF)

Figure S5 Alteration of miRNA gene expression in IBD tissues: sub-chromosomal localization of the affected loci. Chromosomal bands where 2 (arrowheads) or more (squares) miRNA genes with altered expression colocalize are shown. Grey and light-red symbols represent loci where IBD susceptibility has yet been demonstrated by genetic means and previously unidentified loci, respectively.
(0.12 MB TIF)

Table S1 miRNA expression in left and right colon of healthy individuals. Relative miRNA expression (10×log102^∼ΔCT^; see Materials and Methods) was calculated in the right and left colons independently. Note that miRNA expression was similar in both segments of the colon.
(0.02 MB XLS)

Table S2 Characteristics of patients with CD or UC and of healthy control individuals. * Sex: F : female/M : male; + disease location: C: colon/IC: ileocolonic/C+AP: colon and anoperineal lesions/R: right colon/S: sigmoid colon/LC: left colon; # Current treatment : CS: steroids/5 ASA: 5 aminosalicylates/AZA: azathioprine/IFX: infliximab/MTX: methotrexate.
(0.01 MB XLSX)

Table S3 Alterations of miRNA expression in UC patients. Relative miRNA expression was computed vs. that measured in healthy controls and expressed as 10×log102^−ΔΔCT^. Are only listed the miRNAs with relative expressions >7 or <−7 in non-inflamed UC tissues. When adequate, alteration of expression in inflamed UC is also mentioned. Access_N°, MIMAT identification number; Mean ± Sem (5–8 patients). Statistical significance (p values) was calculated relative to healthy control tissue using the non-parametric Mann-Whitney test. Bolded, miRNA with statistically significant dysregulation of expression in both quiescent and inflamed UC.
(0.03 MB XLS)

Table S4 Alterations of miRNA expression in CD patients. Relative miRNA expression was computed vs. that measured in healthy controls and expressed as 10×log102^−ΔΔCT^. Are only listed the miRNAs with relative expressions >7 or <−7 in non-inflamed CD tissues. When adequate, alteration of expression in inflamed CD is also mentioned. Access_N°, MIMAT identification number; Mean ± Sem (5–8 patients). Statisticalsignificance (p values) was calculated relative to healthy control tissue using the non-parametric Mann-Whitney test. Bolded, miRNA with statistically significant dysregulation of expression both in quiescent and in inflamed CD.
(0.04 MB XLS)

Table S5 Shared alterations of miRNA expression in UC and CD patients. Relative miRNA expression was computed vs. that measured in healthy controls. miRNA with shared and significant overexpression (10×log102^−ΔΔCT^>7) both in non-inflamed UC and in non-inflamed CD tissues are listed. Access_N°, MIMAT identification number; Mean ± Sem (5–8 patients). Italics (2 lower rows), miRNA that are not overexpressed in inflamed UC (mir-196a) or CD (mir-30c).
(0.02 MB XLS)

Table S6 Compilation of the characteristics of the miRNA with significantly altered expression in quiescent UC and CD colonic mucosa. Access_N°, MIMAT identification number; Gene-Id, miRNA gene identification number; Coordinates, coordinate of the miRNA gene on the chromosome [strand] (from http://www.mirbase.org/cgi-bin/mirna_summary.pl?org=hsa); Band, Chromosomal band where the miRNA gene is localized; Gene Context, presence or absence (intergenic) of overlap between the miRNA gene and another gene either on the same or on the opposite strand.
(0.05 MB XLS)

Table S7 Compilation of the sub-chromosomal regions where acknowledged IBD-susceptibility loci and miRNA genes with altered expression colocalize. Chromosomal locations where colocalize miRNA genes with altered expression relative to healthy controls in quiescent and/or inflamed UC or CD tissues and (i) acknowledged IBD susceptibility loci or (ii) replicated sub-chromosomal regions identified in GWAS are listed. The location of each IBD susceptibility loci is reminded. Gene_Id, miRNA gene identification number; Locus, chromosomal band where the miRNA gene is localized; Quiescent, non-inflamed; Both, non-inflamed and inflamed; +/−, +: overexpression; −: underexpression.
(0.01 MB XLSX)

Table S8 Alteration of miRNA expression in the colonic mucosa of UC and CD patients: in silico characterization of target transcripts. The exhaustive list of genes which are putatively targeted by the subset of 8 miRNAs that share common dysregulated overexpression in both UC and CD was downloaded from the PITA catalog of predicted human microRNA targets (http://genie.weizmann.ac.il/pubs/mir07/mir07_data.html). The algorithm makes use of the parameter-free model for miRNA-target interaction described by Kertesz et al. (2007). Genes involved in a common biological process are listed together. Bold characters: strictly down regulated genes (the 3′-UTR of which interacts only with up-regulated miRNA(s))
(0.08 MB XLS)
