# Supplementary figures and images for: Identification of Restricted Subsets of Mature microRNA Abnormally Expressed in Inactive Colonic Mucosa of Patients with Inflammatory Bowel Disease
Source: PLoS One. 2010 Oct 5;5(10):e13160. doi: 10.1371/journal.pone.0013160 (PMC2950152; doi:10.1371/journal.pone.0013160)

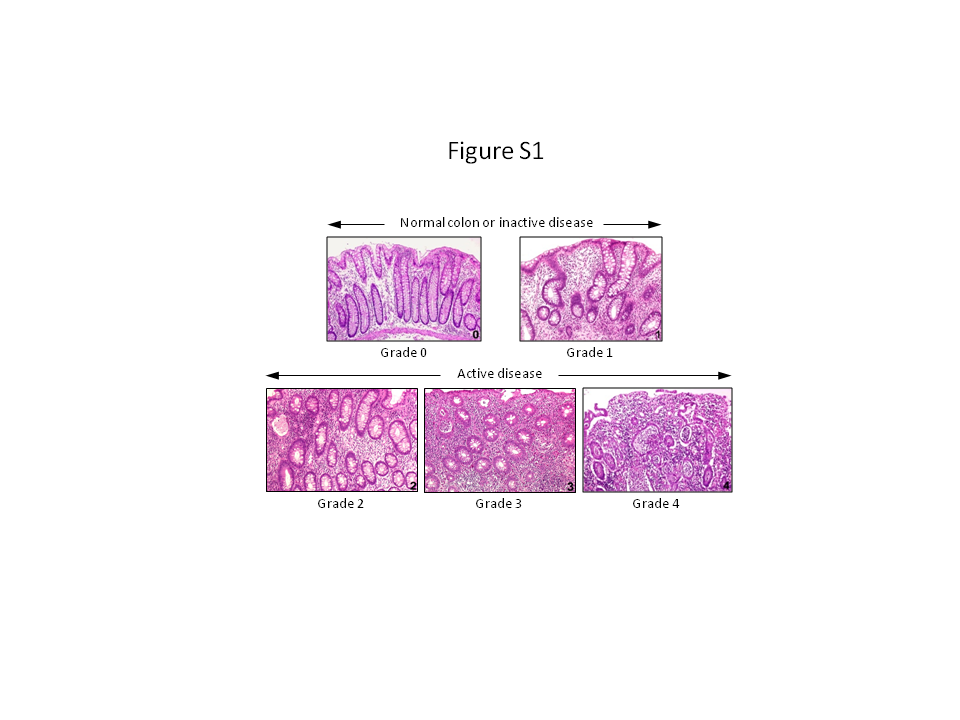

Supplement: Figure S1 — Histological grading of disease activity in colonic biopsies. Hematoxylin and eosin staining of biopsies from non-inflamed and inflamed colonic mucosa (see Materials and Methods for details on histological grading). Note the progressive loss of intestinal epithelium with increasing grade of the disease (2–4) and the concomitant increase in the severity of inflammation/infiltration. Magnification, ×100. (0.35 MB TIF) [file pone.0013160.s001.tif]

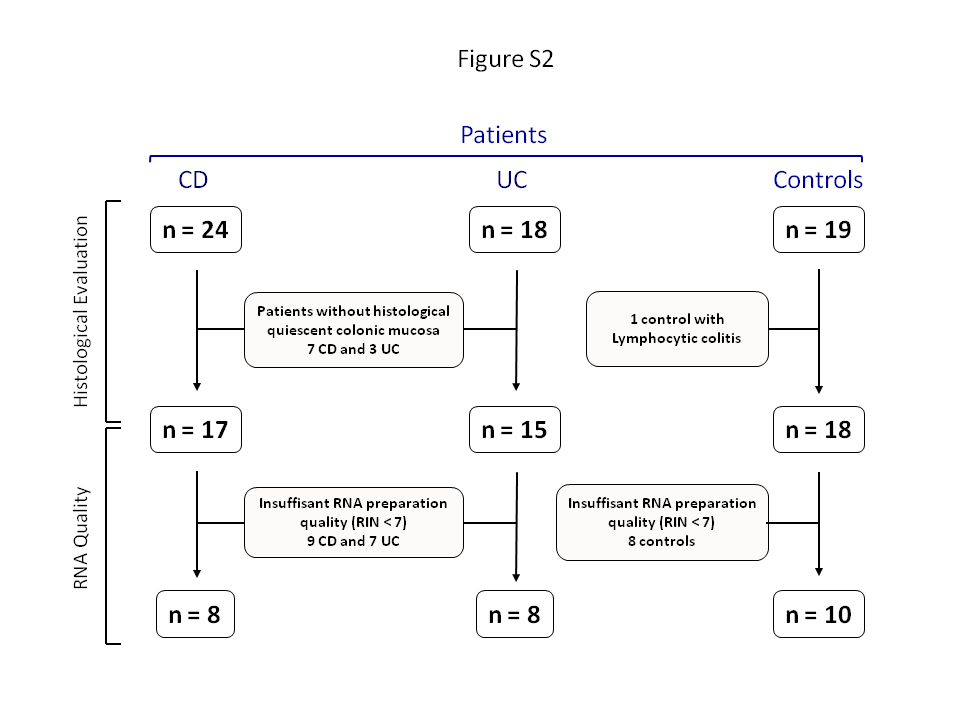

Supplement: Figure S2 — Flow chart of sample selection. In order to exclude any bias in homogeneity among samples, biopsies of patients with endoscopically quiescent, but histologically active colonic mucosa were excluded. The histological dichotomy (grades 0–1 vs. 2–4) was strictly followed to study the alteration in miRNA gene expression. Similarly, 1 control patient with lymphocytic colitis was excluded. In all cases, RNA preparations of low integrity (RIN<7) were discarded. (0.09 MB TIF) [file pone.0013160.s002.tif]

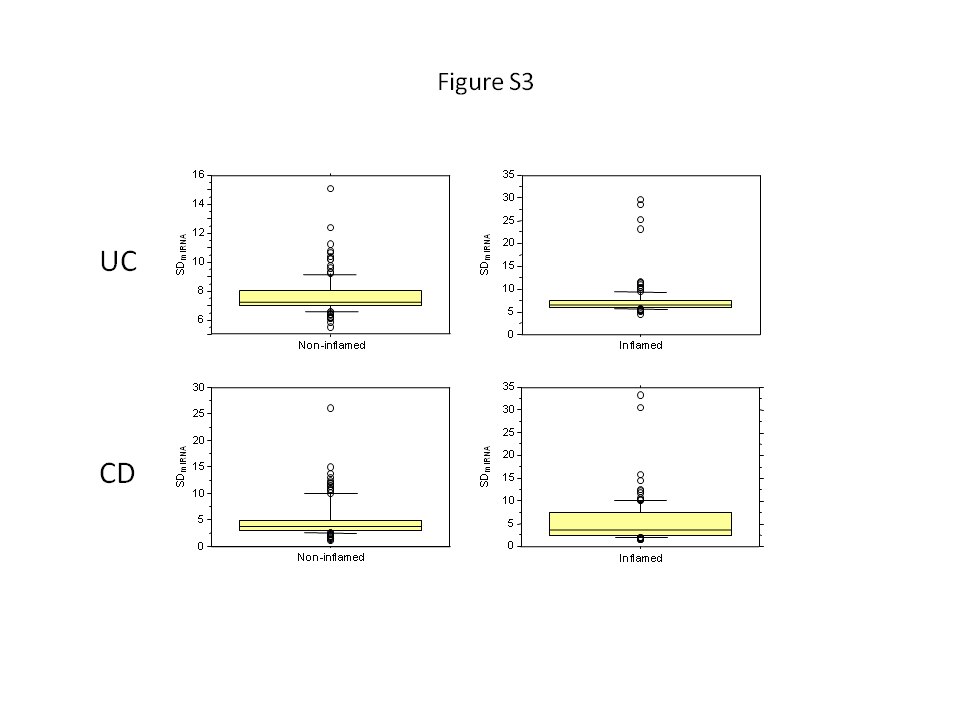

Supplement: Figure S3 — Alteration of miRNA gene expression in non-inflamed and inflamed UC and CD tissues : Box-whisker plot analysis of standard deviations. miRNA expression was measured in non-inflamed and inflamed colonic mucosa obtained from patients with UC and CD (8 patients/IBD) and computed vs. that measured in healthy controls. The mean and standard deviation (SDmiRNA) of relative miRNA expression were then calculated for every miRNA, independently in each IBD for each state of the disease. SDmiRNA were then plotted as box-whisker plots (box, 25–75%; whisker, 10–90%; line, median) (1). 1 Tukey, J. W. (1977) in Exploratory Data Analysis (Addison-Wesley, Reading, MA), pp. 39–43. (0.06 MB TIF) [file pone.0013160.s003.tif]

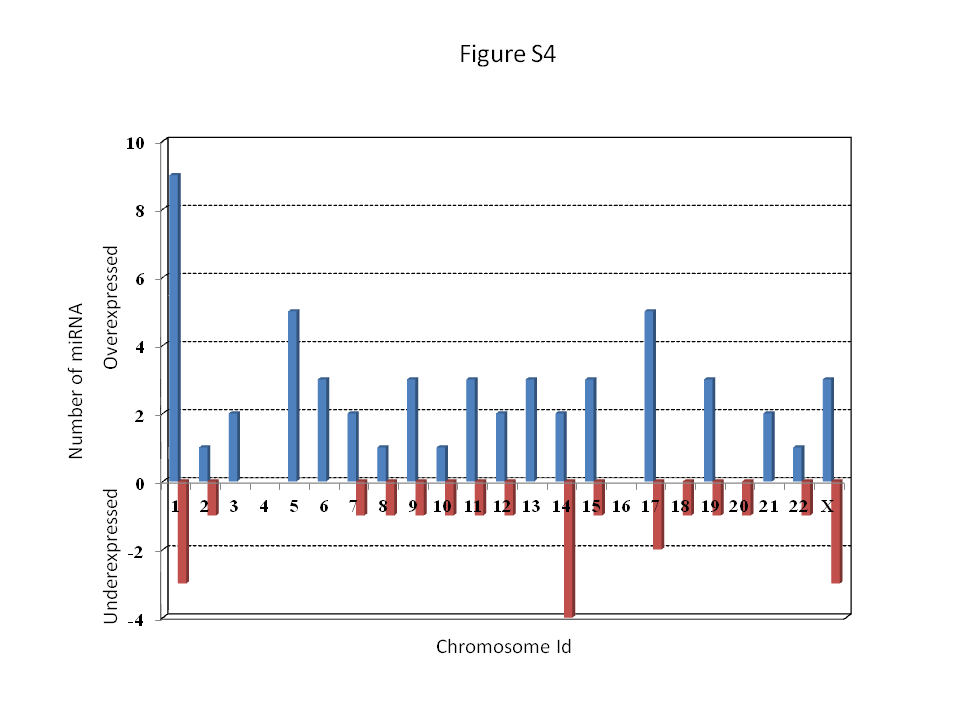

Supplement: Figure S4 — Overview of the chromosomal distribution of miRNA genes with altered expression in IBD tissues: The total number of miRNA genes with altered expression was determined by chromosome. Negative and positive ordinates stand of over and under -expression, respectively. (0.10 MB TIF) [file pone.0013160.s004.tif]

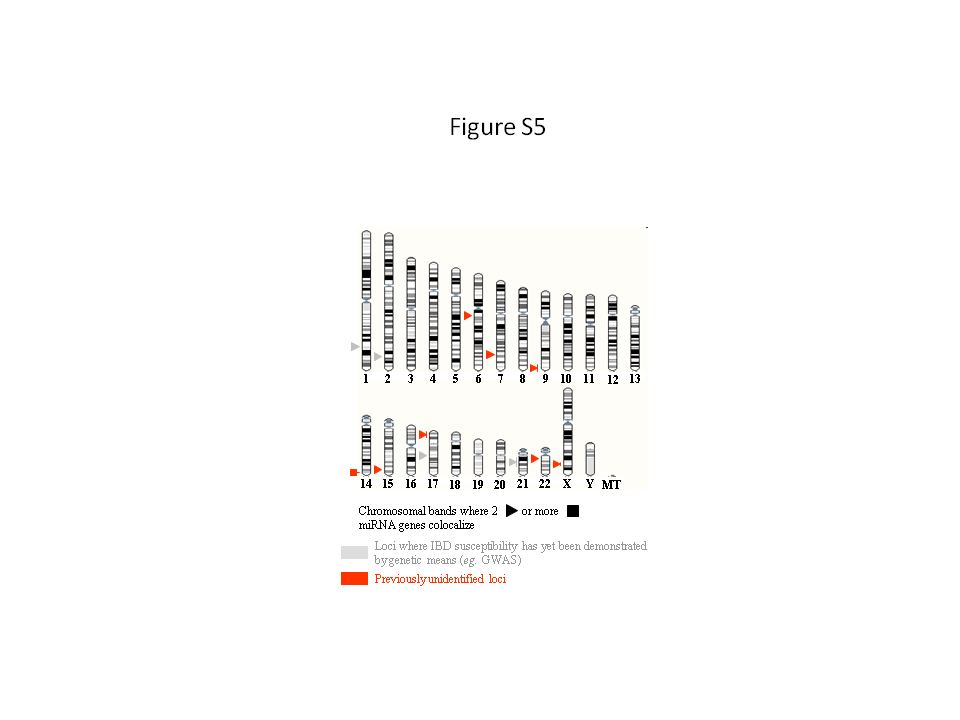

Supplement: Figure S5 — Alteration of miRNA gene expression in IBD tissues: sub-chromosomal localization of the affected loci. Chromosomal bands where 2 (arrowheads) or more (squares) miRNA genes with altered expression colocalize are shown. Grey and light-red symbols represent loci where IBD susceptibility has yet been demonstrated by genetic means and previously unidentified loci, respectively. (0.12 MB TIF) [file pone.0013160.s005.tif]
